# Annual Meeting of the Medical Association of Southern Central New York

**Published:** 1849-08

**Authors:** C. Green

**Affiliations:** Homer


*Annual Meeting of the Medical Association of Southern Central New York.*

We take the liberty of inserting the following extract from a letter by an esteemed correspondent, although not, perhaps, intended by the writer for publication:

HOMER, June 19th, 1849.

DR. FLINT,

DEAR SIR:—It may be a matter of interest to you, as a medical journalist, to know something of the results of the 2nd. Annual Meeting of the Medical Association of Southern Central New York, which took place at Cortlandville, on Tuesday, the 6th inst. Tuesday, P. M., and Wednesday, A. M., were occupied in the relation of cases, and free conversation on them. Numerous facts and observations of a practical character were brought out during narrations. Wednesday, P. M., was taken up in reading a full report of the Committee on Preliminary Education, and also by the reading of an Essay. Thursday A. M. was occupied in the reading of Essays. The afternoon was spent in listening to able reports on epidemic cerebro-spinal meningitis, and on (what was termed) "epidemic fever," or "epidemic petechial fever." These reports, with other portions of the transactions, will, probably, soon be published in due form. DR. BROOKS, of Binghampton, was elected PRESIDENT: DR WALDO, of Berkshire, and DR. WILCOX, of Elmira, VICE

Presidents; Dr. C. Green, of Homer, Recording Secretary; Dr. J. H. Jerome, of Trumansburg, Assistant Recording Secretary; Dr. C. E. Washburn, of Binghampton, Corresponding Secretary. The next Annual Meeting will be held at Elmira. The meeting was characterized by great harmony—vain disputes, about questions of no profit, were omitted, and the time was fully occupied with effective, practical business. We cannot avoid the conclusion, that the direct tendency of our Association is the professional elevation of its members. No one that has ever actively engaged in its business transactions, will voluntarily yield the privilege of membership.

Yours truly,

C. GREEN.

